# Supplementary material for: Novel Rubber Composites Based on Copper Particles, Multi-Wall Carbon Nanotubes and Their Hybrid for Stretchable Devices
Source: Polymers (Basel). 2022 Sep 7;14(18):3744. doi: 10.3390/polym14183744 (PMC9505250; doi:10.3390/polym14183744)
Supplement: Supplementary file 1 [file polymers-14-03744-s001.zip › polymers-1875812-supplementary.pdf]

polymers

MDPI

Supporting Information: Novel Rubber Composites Based on Copper Particles, Multi-Wall Carbon Nanotubes and Their Hybrid for Stretchable Devices

Vineet Kumar, Siraj Azam, Md. Najib Alam, Won-Beom Hong, Sang-Shin Park

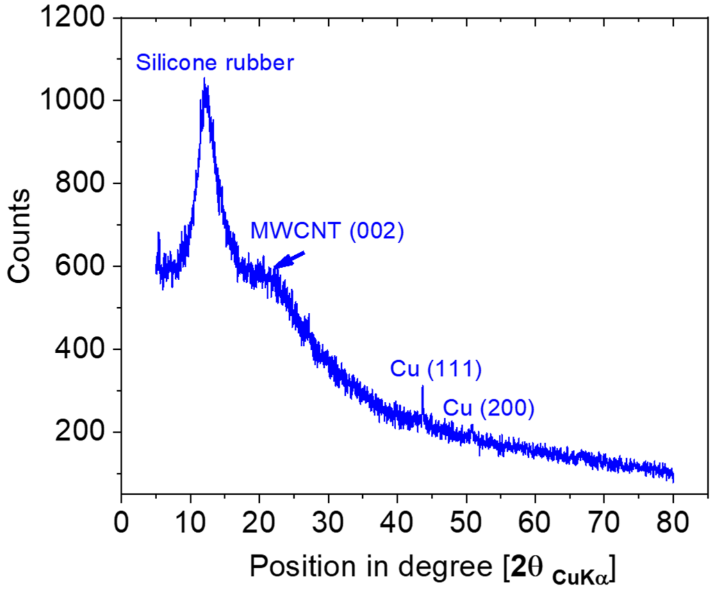

Figure S1. XRD of the hybrid composite containing copper and MWCNT particles.
